# Can combined intracavitary/interstitial approach be an alternative to interstitial brachytherapy with the Martinez Universal Perineal Interstitial Template (MUPIT) in computed tomography-guided adaptive brachytherapy for bulky and/or irregularly shaped gynecological tumors?

**DOI:** 10.1186/s13014-014-0222-6

**Published:** 2014-10-16

**Authors:** Takahiro Oike, Tatsuya Ohno, Shin-ei Noda, Hiroki Kiyohara, Ken Ando, Kei Shibuya, Tomoaki Tamaki, Yosuke Takakusagi, Hiro Sato, Takashi Nakano

**Affiliations:** Department of Radiation Oncology, Gunma University Graduate School of Medicine, 3-39-22, Showa-machi, Maebashi, Gunma 371-8511 Japan

## Abstract

**Background:**

Interstitial brachytherapy (ISBT) is an optional treatment for locally advanced gynecological tumours for which conventional intracavitary brachytherapy (ICBT) would result in suboptimal dose coverage. However, ISBT with Martinez Universal Perineal Interstitial Template (MUPIT), in which ~10-20 needles are usually applied, is more time-consuming and labor-intensive than ICBT alone, making it a burden on both practitioners and patients. Therefore, here we investigated the applicability of a combined intracavitary/interstitial (IC/IS) approach in image-guided adaptive brachytherapy for bulky and/or irregularly shaped gynecological tumours for which interstitial brachytherapy (ISBT) was performed.

**Methods:**

Twenty-one consecutive patients with gynecological malignancies treated with computed tomography-guided ISBT using MUPIT were analyzed as cases for this dosimetric study. For each patient, the IC/IS plan using a tandem and 1 or 2 interstitial needles, which was modeled after the combined IC/IS approach, was generated and compared with the IS plan based on the clinical ISBT plan, while the IC plan using only the tandem was applied as a simplified control. Maximal dose was prescribed to the high-risk clinical target volume (HR-CTV) while keeping the dose constraints of D_2cc_ bladder < 7.0 Gy and D_2cc_ rectum < 6.0 Gy. The plan with D90 HR-CTV exceeding 6.0 Gy was considered acceptable.

**Results:**

The average D90 HR-CTV was 77%, 118% and 140% in the IC, IC/IS and IS plans, respectively, where 6 Gy corresponds to 100%. The average of the ratio of D90 HR-CTV to D_2cc_ rectum (gain factor (GF) _rectum_) in the IC, IC/IS and IS plans was 0.8, 1.3 and 1.5 respectively, while GF_bladder_ was 0.9, 1.4 and 1.6, respectively. In the IC/IS plan, D90 HR-CTV, GF_rectum_ and GF_bladder_ exceeded 100%, 1.0 and 1.0, respectively, in all patients.

**Conclusions:**

These data demonstrated that the combined IC/IS approach could be a viable alternative to ISBT for gynecological malignancies with bulky and/or irregularly shaped tumours.

**Electronic supplementary material:**

The online version of this article (doi:10.1186/s13014-014-0222-6) contains supplementary material, which is available to authorized users.

## Background

Interstitial brachytherapy (ISBT) is a choice of curative treatment for locally advanced gynecological tumours with extension to the lateral parametria and/or lower vaginal wall, for which intracavitary brachytherapy (ICBT) would result in suboptimal dose coverage [1]. Accordingly, we have been treating patients with locally advanced cervical tumours and other gynecological malignancies using in-room and in-situ computed tomography (CT)-guided adaptive high dose rate (HDR) ISBT with Martinez Universal Perineal Interstitial Template (MUPIT, Nucleotron) [2]. However, ISBT with MUPIT, in which ~10-20 needles are usually applied, is more time-consuming and labor-intensive than ICBT alone, making it a burden on practitioners. Moreover, patients receiving this treatment also must bear the hardship of keeping interstitial needles implanted for several days. Besides, facilities at which ISBT is routinely performed are limited in Japan [3]. These aspects have prompted us to explore alternative treatment strategies for bulky and/or irregularly shaped tumours.

The recent development of technologies in image-guided adaptive brachytherapy (IGABT) has enabled the integration of magnetic resonance imaging (MRI) or CT images into the treatment planning process [4-6]. This has resulted in the adaptive target concept, with dose volume assessment balancing constraints for the clinical target volume (CTV) and organs at risk (OARs). In 2011, Pötter *et al*. reported the long-term results of 156 cervical cancer patients treated with chemoradiotherapy, demonstrating an overall 3-year local control rate of 92% for locally advanced tumours (>5 cm) treated with the MRI-guided combined intracavitary/interstitial (IC/IS) approach [7]. This indicates that the image-guided combined IC/IS approach with a few interstitial needles could be a viable alternative to ISBT with MUPIT in the treatment of bulky and/or irregularly shaped tumours. Therefore, here we performed a dosimetric study to explore the applicability of the CT-guided combined IC/IS approach in patients with gynecological malignancies with bulky and/or irregularly shaped tumours, who were actually treated with CT-guided ISBT with MUPIT.

## Methods

Twenty-one consecutive patients with gynecological malignancies treated with CT-guided adaptive HDR ISBT with MUPIT using iridium-192 source at Gunma University Hospital between April 2008 and February 2013 were selected by IRB-approved retrospective chart review. Fourteen of them (67%) were cervical cancer patients. The rest included vaginal cancer (3 patients), uterine endometrioid cancer (3 patients) and ovarian cancer (1 patient). Among them, 10/14, 1/3, 3/3 and 1/1 patients with cervical, vaginal, uterine endometrioid and ovarian cancer had recurrent tumours. Although these cases were heterogeneous in terms of primary tumour site and whether they were primary diseases or recurrences, they had the commonality of developing bulky and/or irregularly shaped tumours in the uterine cervix or vaginal stump and/or parametrium. Hence, they were considered to be favorable hypothetical cases of locally advanced cervical cancer, which has the indication for the IC/IS approach in the clinic. For example, a recurrent ovarian cancer case showed an intrapelvic tumour with bilateral parametrial involvement predominantly in the right side (Additional file 1: Figure S1). Average ± standard deviation (SD) of high-risk CTV (HR-CTV) in the present study was 60 ± 32 cm^3^, which was similar to the values of patients with locally advanced cervical cancer treated with combined IC/IS by the Utrecht group (66 ± 34 cm^3^) [8]. Moreover, in our study, the distance between the tandem and the outer contour of HR-CTV was 30 mm or greater in all cases. The Utrecht group employed a distance of 30 mm as cutoff value for adding interstitial needles to the usual applicator consisting of the tandem and ovoids in IGABT for locally advanced cervical cancer [9]. Accordingly, the Vienna group reported that a distance of 25 mm in the plane of point A and that of 35 mm at ring level could be covered by sufficient dose using only the intracavitary part of the Vienna applicator [10]. Together, these data suggest that the cases selected in the current study were typical of extended tumours for which ISBT is recommended.

Application of the tandem, an endovaginal cylinder with MUPIT and interstitial needles was performed under in-room and in-situ CT guidance. Detailed procedure of the actually performed ISBT was previously described [2]. Target delineation was performed on CT images taken by an in-room CT scanner with reference to MRI images taken just before brachytherapy. The contours of HR-CTV, rectum and bladder were delineated according to the GEC-ESTRO recommendations [4].

**Figure 1:**
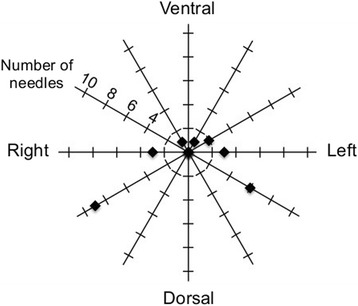
**Frequency of interstitial needles used in the IC/IS plan per needle location.** Relative locations of the needles to the tandem in a transverse plane were plotted in steps of 30 degrees. Twenty-five needles in 21 patients were used.

We aimed to assess the applicability of a plan using the tandem and a few interstitial needles (IC/IS plan) as a viable alternative to the actually performed ISBT plan using MUPIT (IS plan). For this purpose, the IC/IS plan was generated on the CT image used for the actual ISBT treatments in each patient using the PLATO Brachytherapy Planning System (ver.14.3.6, Nucletron) or Oncentra (ver.4, Nucletron). For the IC/IS plan, the number of interstitial needles used for source loading was set at 1 or 2, taking the burden of needle application for the practitioners into consideration. Interstitial needles located at the edge of the extended part of HR-CTV were selected from needles implanted with MUPIT (9–18 needles; median, 14) based on visual inspection. One needle was used in 17 cases with unilaterally extended tumours, while 2 needles were selected in 4 cases with bilaterally extended tumours. Needles implanted in laterodorsal positions were most frequently selected (Figure 1), consistent with a previous report showing 95% of locally advanced cervical cancer cases treated with the Utrecht applicator received needle implantation in laterodorsal positions [8]. This suggests the authenticity of the positions of the needles selected in the current study as those used for the combined IC/IS approach. The IC plans, with only the tandem used for source loading, were generated as simplified controls since treatment plans with ovoids or a ring applicator with a tandem have large diversity in the spatial disposition of the applicators.

In the treatment planning for each of the IC, IC/IS and IS plans, the highest possible dose was prescribed to HR-CTV while keeping the dose constraints for rectum and bladder. Comparison of dose volume parameters for HR-CTV between the plans while keeping the same dose constraints for the OARs helps clarify the dosimetric gain of the plans. We have previously investigated late rectal complications in cervical cancer patients treated with EBRT plus ICBT, showing that the average total EQD2 (α/β = 3.0) for D_2cc_ rectum in cases with late rectal complications and those without was 69 and 57 Gy, respectively. Based on these findings, here we set the dose constraint for rectum at D_2cc_ < 6 Gy, which results in total EQD2 of 59 Gy when the Japanese standard treatment regimen for advanced cervical cancer (30 Gy/15 fractions of whole pelvic irradiation followed by 20 Gy/10 fractions of EBRT with midline block and 4 sessions of ICBT) was employed [11]. There has been no concrete evidence so far regarding the dose constraint for bladder using the Japanese treatment regimen. However, several European groups have revealed that the tolerability of bladder to radiotherapy is relatively higher than that of rectum [7,8]. In reference to their data, we set the dose constraint for bladder at D_2cc_ < 7 Gy.

D90, the most significant dose volume parameter for local control in IGABT for cervical cancer [12], was used to assess HR-CTV coverage. We previously reported DVH analysis of ICBT for cervical cancer, in which CT images taken just before ICBT were superimposed on the 3-dimensional treatment plan of ICBT [13]. The study demonstrated that ′tumour volumes′ almost corresponding to the current HR-CTV in GEC-ESTRO recommendations [5] receiving less than a total of 24 Gy in 4 fractions were significantly larger in patients with local recurrence than in those with local control. Based on these data, here, a plan showing D90 HR-CTV higher than 6 Gy while keeping the dose constraints for the OARs was considered acceptable.

To clearly understand the dosimetric gain of a plan, gain factor (GF) was defined as the ratio of D90 HR-CTV to D_2cc_ of rectum and bladder, respectively: GF_rectum_ = D90 HR-CTV/D_2cc_ rectum, GF_bladder_ = (D90 HR-CTV/D_2cc_ bladder) × 7/6. In the present study, 6 Gy for D_2cc_ rectum and 7 Gy for D_2cc_ bladder were employed as dose constraints, and the plan showing D90 HR-CTV higher than 6 Gy while keeping these dose constraints was considered acceptable. Hence, with regard to rectum, GF_rectum_ of a ″borderline-acceptable″ plan showing D90 HR-CTV of 6 Gy and D_2cc_ rectum of 6 Gy becomes 1.0, favorably. However, in the case of bladder, GF_bladder_ of a ″borderline-acceptable″ plan showing D90 HR-CTV of 6 Gy and D_2cc_ bladder of 7 Gy becomes 0.86 (i.e., 6/7). In order to make this value 1.0, the coefficient ″7/6″ was used in calculating GF_bladder_. Together, a GF higher than 1.0 indicates that the plan has the potential of being definitive and acceptable in terms of late toxicities for rectum or bladder. The treatment plans were confirmed by two radiation oncologists (T. Oike and T. Ohno).

D98 and V_6Gy_ of HR-CTV were also analyzed for target coverage, while V_12Gy_ HR-CTV was examined for high dose volume [5]. For inter-comparison of plans with different dose prescriptions, V_6Gy_ and V_12Gy_, the volumes receiving at least 6 and 12 Gy, were selected as alternatives for V100 and V200, respectively. Differences in DVH parameters were evaluated for significance by paired *t*-test using StatMateIII (ver. 3.17, ATMS). A *P* value < 0.05 was considered significant.

Of note, in the present study, the cases were analyzed simply as samples for dosimetric comparison of the treatment plans to test the applicability of the combined IC/IS approach for bulky and/or irregularly shaped tumours. Thus, here we do not aim to discuss the curability of these cases in light of the actually performed treatments. Accordingly, the clinical information, including treatment history, simultaneous use of EBRT and total EQD2, is not presented.

## Results and discussion

**Figure 2:**
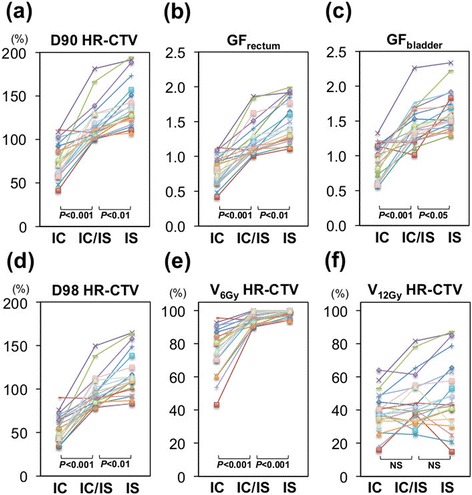
**DVH parameters in the IC, IC/IS and IS plans in all patients. (a)** D90 HR-CTV, **(b)** GF_rectum_, **(c)** GF_bladder_, **(d)** D98 HR-CTV, **(e)** V_6Gy_ HR-CTV, **(f)** V_12Gy_ HR-CTV. D90 and D98 HR-CTV were described in “%”, where 6 Gy corresponds to 100%. NS, not statistically significant.

**Figure 3:**
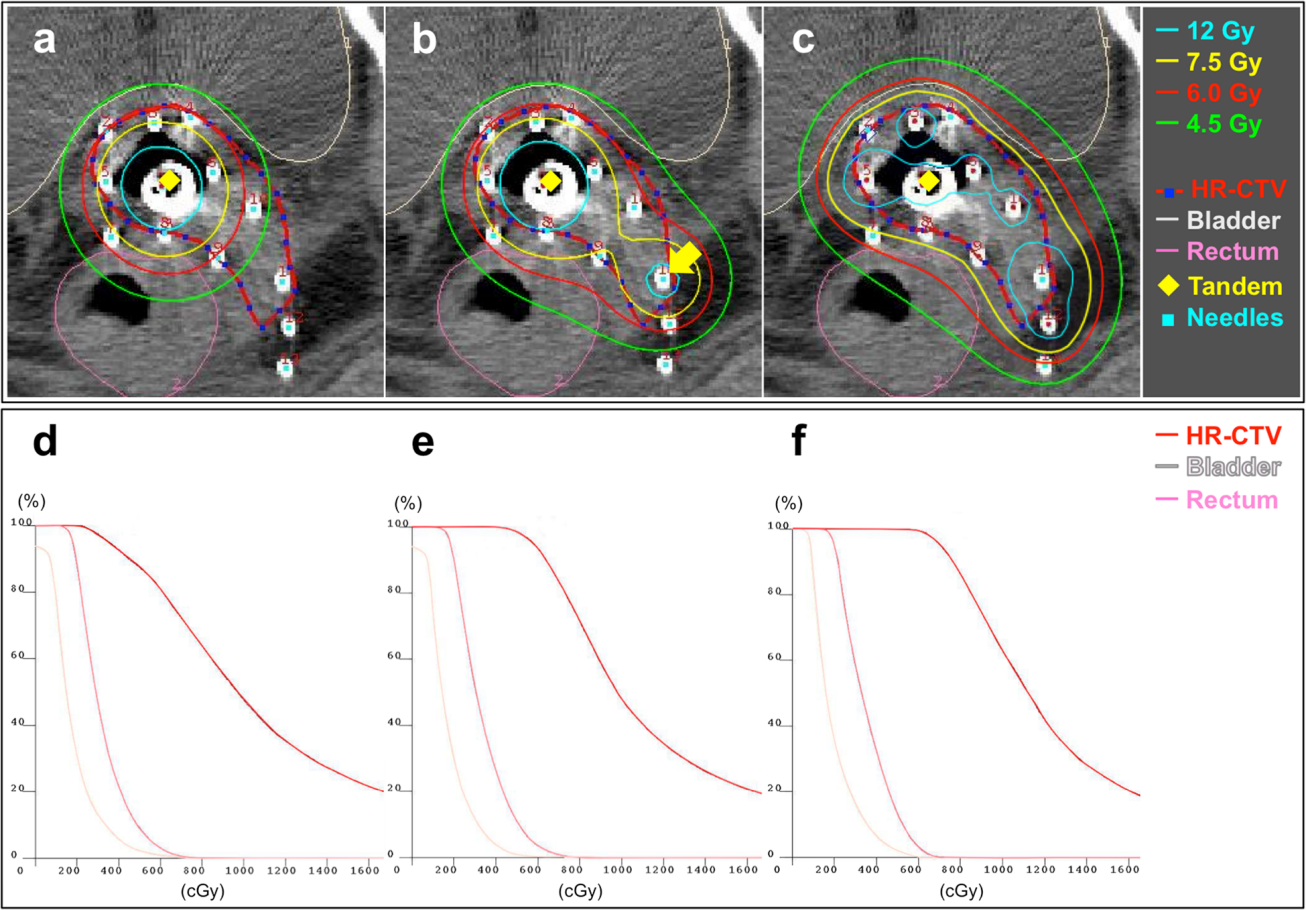
**Representative dose distributions in transverse planes (a-c) and DVH curves (d-f) provided by the IC plan (a, d), the IC/IS plan with 1 needle (b, e), and the IS plan (c, f).** The case is a 55-year-old patient with newly diagnosed cervical cancer, FIGO stage IIIB, treated with concurrent chemoradiotherapy using weekly cisplatin. **(a)** Source loading via only the tandem shows insufficient coverage of HR-CTV (D90, 4.6 Gy; D98, 2.9 Gy; V_6Gy_, 81%). **(b)** Addition of source loading via 1 needle (arrow) in expanded tumour in the left laterodorsal direction improved HR-CTV coverage (D90, 6.3 Gy; D98, 5.1 Gy; V_6Gy_, 93%). **(c)** Usage of the tandem and all 12 needles for source loading further improved HR-CTV coverage (D90, 7.6 Gy; D98, 6.5 Gy; V_6Gy_, 100%).

The IC plans with only the tandem resulted in insufficient HR-CTV coverage as expected (Figure 2a-e, Figure 3a, Table 1). D90 HR-CTV was below 100% (i.e., 6.0 Gy) in 81% (17/21). GF_rectum_ and GF_bladder_ were below 1.0 in 81% (17/21) and 52% (11/21), respectively. Interestingly, the addition of one or two needles to the IC plan (i.e., the IC/IS plan) dramatically improved the HR-CTV coverage (Figure 2a-e, Figure 3b, Table 1). The D90 HR-CTV, GF_rectum_, GF_bladder_, D98 HR-CTV and V_6Gy_ HR-CTV of the IC/IS plan was significantly higher than that of the IC plan (*P* < 0.001 in all parameters). Of note, D90 HR-CTV, GF_rectum_ and GF_bladder_ exceeded 100% (i.e., 6.0 Gy), 1.0 and 1.0, respectively, in all cases. These data indicate that the combined IC/IS approach can achieve satisfactory dose distribution with acceptable HR-CTV coverage and tolerable sparing of rectum and bladder in bulky and/or irregularly shaped tumours for which ISBT was performed.

**Table 1:** **DVH parameters in IC, IC/IS and IS plans**

	**HR-CTV**				**Gain factor**	
	**D90 (%)**	**D98 (%)**	**V6Gy (%)**	**V12Gy (%)**	**Rectum**	**Bladder**
IC plan	77 (20)	53 (15)	77 (14)	39 (12)	0.8 (0.2)	0.9 (0.2)
IC/IS plan	118 (22)	97 (18)	95 (3.5)	45 (16)	1.3 (0.3)	1.4 (0.3)
IS plan	140 (25)	115 (25)	98 (2.1)	50 (21)	1.5 (0.3)	1.6 (0.3)

Averages (SD) are shown.

In our IC/IS model with a few needles, the reproducible placement of needles to the most appropriate positions of HR-CTV is required to achieve favorable dose distribution. For this purpose, the Vienna (I) and Utrecht applicators dedicated to the combined IC/IS approach would become effective tools [10,14], although the lateral dimensions of the extended isodose lines are limited after adding needles. Furthermore, exploration to identify the optimal treatment plan (i.e., optimal number and position of needles) for balancing satisfactory dose distributions and clinical burden should be conducted in the near future.

Our data and those of others indicate that needles in laterodorsal positions are most frequently used for source loading in the combined IC/IS approach (Figure 1) [8]. Meanwhile, laterodorsal contours of HR-CTV tend to be overestimated by CT compared with MRI, which is a major advantage of MRI in discrimination of tumours and adjacent normal tissue [5,15]. In this regard, it can be said that our study has a limitation in comparing the target coverage in the laterodorsal directions between ISBT with MUPIT and the combined IC/IS approach. Thus, an analysis in the same way as the present study and using MRI-based treatment plans should be carried out. Thus, to validate our results of the MRI-guided combined IC/IS approach, we are preparing to introduce a treatment planning system in which MRI images can be directly registered onto CT images, which should further improve the identification of HR-CTV.

The IS plans achieved more conformal coverage of HR-CTV than the IC/IS plans, leading to further improvement of HR-CTV coverage and GFs compared to the IC/IS plans (Figure 2a-e, Figure 3c, Table 1). The D90 HR-CTV, GF_rectum_, GF_bladder_, D98 HR-CTV and V_6Gy_ HR-CTV values of the IS plan were significantly higher than those of the IC/IS plan (*P* < 0.01, *P* < 0.01, *P* < 0.05, *P* < 0.01 and *P* < 0.001, respectively). In fact, a previous study has pointed out the existence of cases with extremely bulky and/or irregularly shaped tumours in which even the combined IC/IS approach resulted in insufficient coverage for HR-CTV [8]. Together, ISBT with MUPIT can still be advantageous for such cases.

The number and geometrical distribution of interstitial needles affect the conformality of HR-CTV coverage. Therefore, the conformality of HR-CTV coverage is of particularly high importance in the combined IC/IS approach using less needles than ISBT. Meanwhile, several indices such as the conformality index (COIN) to evaluate the conformality of target coverage in brachytherapy have been proposed [16]. Interestingly, in the setting of the current study where maximal dose was prescribed to HR-CTV while keeping the dose constraint for the rectum (6 Gy) and bladder (7 Gy), COIN becomes identical to V_6Gy_ HR-CTV. As shown in Figure 2e, V_6Gy_ HR-CTV in the IC/IS plans has been dramatically improved compared to that in the IC plans, nearing that in the IC/IS plans. These data indicate that the IC/IS approach improves its conformality compared with the IC approach, and is close to ISBT in terms of the conformality of HR-CTV coverage in bulky and/or irregularly shaped gynecological tumours.

On the other hand, there was no significant difference in V_12Gy_ among the three plans (Figure 2f, Table 1). Although V150 and V200 are recommended as DVH parameters for the high dose volume by GEC-ESTRO [11], the clinical significance of the high dose volume has not been elucidated by various IGABT techniques, which certainly warrants further investigation.

The present study has limitations. First, the study design of employing ICBT with a tandem alone as a control is weak and is not suitable for clinical use in locally advanced gynecological tumours. However, there are many types of applicators for ICBT used in combination with a tandem, including ovoids and a ring applicator. In treatment planning using these applicators, large numbers of variables such as three-dimensional spatial disposition of the applicators and dwell time setting make it difficult to standardize the scheme for optimization of a control ICBT plan among patients, leading to the difficulty of evaluating the pure dosimetric gain of additional needles. Furthermore, dosimetric study comparing a combined IC/IS technique using tandem, ovoids and a few interstitial needles with ISBT in the same patient is almost impossible because ovoids are not usually used in ISBT in the clinic. Thus, if we aim to perform such dosimetric study, comparison of the two methods using different patients with different anatomical characteristics in terms of tumours and OARs is inevitable. Such a study design also results in a weak conclusion. Upon taking these issues into consideration, in the present study, we employed the IC plan with only a tandem as a control. The optimal number and spatial disposition of needles in combination with a tandem and other applicators including ovoids should be further investigated. Second, the present study was carried out based on the assumption that the anatomy among the IC, the IC/IS and the IS approach remains the same. However, in the clinical setting, the anatomy, in particular the 3D-configuration of HR-CTV, can be different among the three methods according to the number and distribution of inserted needles. This issue should be carefully considered in the clinical application of the combined IC/IS approach as an alternative to ISBT with MUPIT.

## Conclusions

Here we have demonstrated a treatment plan consisting of a tandem and at most 2 needles, which is a simplified model for the combined IC/IS approach, achieving our dose prescription criteria (i.e., D90 HR-CTV > 6.0 Gy, D_2cc_ rectum < 6.0 Gy and D_2cc_ bladder < 7.0 Gy per fraction) in all 21 consecutive patients with gynecological malignancies actually treated with CT-guided ISBT using MUPIT. This indicates the potential of the combined IC/IS approach as an alternative to ISBT with MUPIT in CT-guided adaptive brachytherapy for bulky and/or irregularly shaped gynecological tumours. Further research to assess the clinical feasibility of the combined IC/IS approach should be carried out with an optimal number and spatial disposition of needles in combination with a tandem and ovoids, and also anatomical change by the insertion of a tandem and needles should be taken into consideration.

## Additional file

Additional file 1: Figure S1 Representative CT image of an implant for the recurrent ovarian cancer case. The tumour shows bilateral parametrial involvement predominant in the right side. An intrauterine tandem and 11 interstitial needles (white dots) were applied.

## References

[1] Nag S, Erickson B, Thomadsen B, Orton C, Demanes JD, Petereit D (2000). The American Brachytherapy Society recommendations for high-dose-rate brachytherapy for carcinoma of the cervix. Int J Radiat Oncol Biol Phys.

[2] Saitoh JI, Ohno T, Sakurai H, Katoh H, Wakatsuki M, Noda SE, Suzuki Y, Shibuya K, Takahashi T, Nakano T (2011). High-dose-rate interstitial brachytherapy with computed tomography-based treatment planning for patients with locally advanced uterine cervical carcinoma. J Radiat Res.

[3] Tomita N, Toita T, Kodaira T, Shinoda A, Uno T, Numasaki H, Teshima T, Mitsumori M (2012). Patterns of radiotherapy practice for patients with cervical cancer in Japan, 2003–2005: Changing trends in the patterns of care process. Int J Radiat Oncol Biol Phys.

[4] Haie-Meder C, Pötter R, Van Limbergen E, Briot E, De Brabandere M, Dimopoulos J, Dumas I, Hellebust TP, Kirisits C, Lang S, Muschitz S, Nevinson J, Nulens A, Petrow P, Wachter-Gerstner N (2005). Gynaecological (GYN) GEC-ESTRO Working Group: Recommendations from Gynaecological (GYN) GEC-ESTRO Working Group (I): concepts and terms in 3D image based 3D treatment planning in cervix cancer brachytherapy with emphasis on MRI assessment of GTV and CTV. Radiother Oncol.

[5] Pötter R, Haie-Meder C, Van Limbergen E, Barillot I, De Brabandere M, Dimopoulos J, Dumas I, Erickson B, Lang S, Nulens A, Petrow P, Rownd J, Kirisits C (2006). GEC ESTRO Working Group: Recommendations from gynaecological (GYN) GEC ESTRO working group (II): concepts and terms in 3D image-based treatment planning in cervix cancer brachytherapy-3D dose volume parameters and aspects of 3D image-based anatomy, radiation physics, radiobiology. Radiother Oncol.

[6] Charra-Brunaud C, Levitchi M, Delannes M: **Dosimetric, clinical results of a French prospective study of 3D brachytherapy for cervix carcinoma.** *Radiother Oncol* 2011, **99:**S57.

[7] Pötter R, Georg P, Dimopoulos JC, Grimm M, Berger D, Nesvacil N, Georg D, Schmid MP, Reinthaller A, Sturdza A, Kirisits C (2011). Clinical outcome of protocol based image (MRI) guided adaptive brachytherapy combined with 3D conformal radiotherapy with or without chemotherapy in patients with locally advanced cervical cancer. Radiother Oncol.

[8] Nomden CN, de Leeuw AA, Moerland MA, Roesink JM, Tersteeg RJ, Jürgenliemk-Schulz IM (2012). Clinical use of the Utrecht applicator for combined intracavitary/interstitial brachytherapy treatment in locally advanced cervical cancer. Int J Radiat Oncol Biol Phys.

[9] Georg P, Lang S, Dimopoulos JCA, Dörr W, Sturdza AE, Berger D, Georg D, Kirisits C, Pötter R (2011). Dose-volume histogram parameters and late side effects in magnetic resonance image-guided adaptive cervical cancer brachytherapy. Int J Radiat Oncol Biol Phys.

[10] Kirisits C, Lang S, Dimopoulos J, Berger D, Georg D, Pötter R (2006). The Vienna applicator for combined intracavitary and interstitial brachytherapy of cervical cancer: Design, application, treatment planning, and dosimetric results. Int J Radiat Oncol Biol Phys.

[11] Kato S, Linh TDN, Ohno T, Nakano T, Kiyohara H, Ohkubo Y, Kamada T (2010). CT-based 3D dose volume parameter of the rectum and late rectal complication in patients with cervical cancer treated with high-dose-rate intracavitary brachytherapy. J Radiat Res.

[12] Dimopoulos JC, Lang S, Kirisits C, Fidarova EF, Berger D, Georg P, Dörr W, Pötter R (2009). Dose-volume histogram parameters and local tumour control in magnetic resonance image-guided cervical cancer brachytherapy. Int J Radiat Oncol Biol Phys.

[13] Terahara A, Nakano T, Ishikawa A, Morita S, Tsuji H (1996). Dose-volume histogram analysis of high dose rate intracavitary brachytherapy for uterine cervix cancer. Int J Radiat Oncol Biol Phys.

[14] Jurgenliemk-Schulz IM, Tersteeg RJ, Roesink JM, Bijmolt S, Nomden CN, Moerland MA, de Leeuw AA (2009). MRI-guided treatment-planning optimisation in intracavitary or combined intracavitary/interstitial PDR brachytherapy using tandem ovoid applicators in locally advanced cervical cancer. Radiother Oncol.

[15] Viswanathan AN, Dimopoulos J, Kirisits C, Berger D, Pötter R (2007). Computed tomography versus magnetic resonance imaging-based contouring in cervical cancer brachytherapy: results of a prospective trial and preliminary guidelines for standardized contours. Int J Radiat Oncol Biol Phys.

[16] Baltas D, Kolotas C, Geramani K, Mould RF, Ioannidis G, Kekchidi M, Zamboglou N (1998). A conformal index (COIN) to evaluate implant quality and dose specification in brachytherapy. Int J Radiat Oncol Biol Phys.

