# Supplementary material for: Can combined intracavitary/interstitial approach be an alternative to interstitial brachytherapy with the Martinez Universal Perineal Interstitial Template (MUPIT) in computed tomography-guided adaptive brachytherapy for bulky and/or irregularly shaped gynecological tumors?
Source: Radiat Oncol. 2014 Oct 16;9:222. doi: 10.1186/s13014-014-0222-6 (PMC4205298; doi:10.1186/s13014-014-0222-6)
Supplement: Additional file 1: Figure S1 — Representative CT image of an implant for the recurrent ovarian cancer case. The tumour shows bilateral parametrial involvement predominant in the right side. An intrauterine tandem and 11 interstitial needles (white dots) were applied. [file 13014_2014_222_MOESM1_ESM.pdf]

Supplementary Figure 1

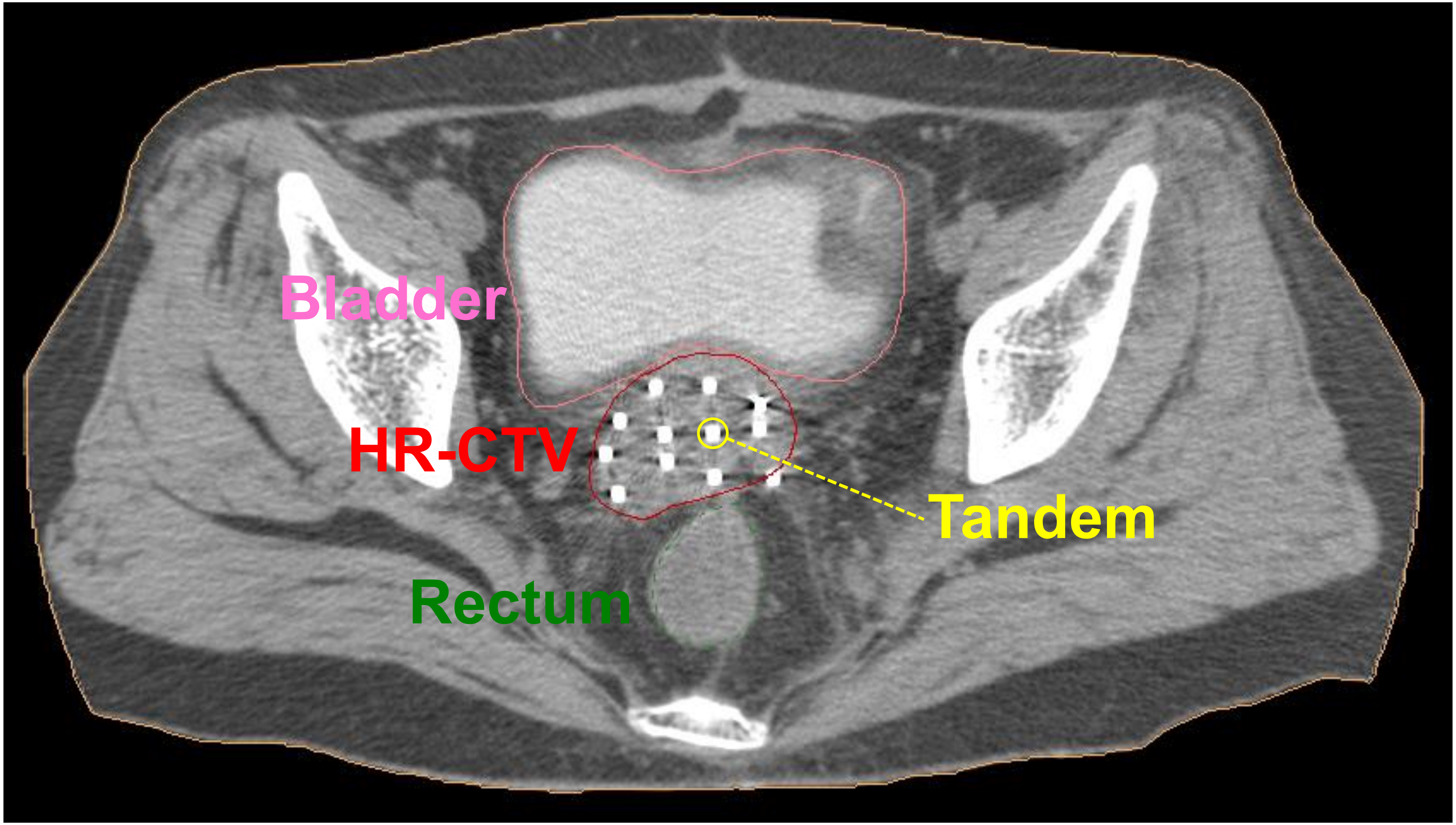

Supplementary Fig. 1. Representative CT image of an implant for the recurrent ovarian cancer case. The tumour shows bilateral parametrial involvement predominant in the right side. An intrauterine tandem and 11 interstitial needles (white dots) were applied.
